# Short-Lived Cages Restrict Protein Diffusion in the Plasma Membrane

**DOI:** 10.1038/srep34987

**Published:** 2016-10-11

**Authors:** Maria Goiko, John R. de Bruyn, Bryan Heit

**Affiliations:** 1 Department of Microbiology and Immunology, The University of Western Ontario, London, Ontario, N6A 5C1 Canada; 2 Department of Physics and Astronomy, The University of Western Ontario, London, Ontario, N6A 3K7 Canada; 3 Centre for Human Immunology, The University of Western Ontario, London, Ontario, N6A 5C1 Canada

## Abstract

The plasma membrane is a heterogeneous environment characterized by anomalous diffusion and the presence of microdomains that are molecularly distinct from the bulk membrane. Using single particle tracking of the C-type lectin CD93, we have identified for the first time the transient trapping of transmembrane proteins in cage-like microdomains which restrict protein diffusion. These cages are stabilized by actin-dependent confinement regions, but are separate structures with sizes and lifespans uncorrelated to those of the underlying actin corral. These membrane cages require cholesterol for their strength and stability, with cholesterol depletion decreasing both. Despite this, cages are much larger in size and are longer lived than lipid rafts, suggesting instead that cholesterol-dependent effects on membrane fluidity or molecular packing play a role in cage formation. This diffusional compartment in the plasma membrane has characteristics of both a diffusional barrier and a membrane microdomain, with a size and lifespan intermediate between short-lived microdomains such as lipid rafts and long-lasting diffusional barriers created by the actin cytoskeleton.

Ever since Singer and Nicholson introduced the fluid mosaic model of the plasma membrane, efforts have been made to understand the diffusion of lipids and proteins within the membrane environment^1^. Early models, such as the hydrodynamic model of Saffman and Delbrück, proposed that diffusion in cellular membranes was Brownian in nature, and therefore that diffusion coefficients would be determined solely by temperature and membrane viscosity^2^. Investigations into membrane diffusion in intact cells revealed that both proteins and lipids have diffusion coefficients an order of magnitude smaller than predicted by Brownian models. Indeed, studies utilizing methods with high temporal resolution revealed that the motion of both lipids and proteins progresses via hop-diffusion, a form of anomalous diffusion driven by the transient trapping of diffusing molecules within confinement regions referred to as “corrals”^3^,^4^,^5^,^6^. In hop diffusion single molecules undergo Brownian diffusion within individual corrals over short time scales, but this molecular motion becomes subdiffusive over intermediate time scales due to frequent collisions with corral “walls” and occasional “hops” which carry the diffusing molecule into neighboring corrals. At longer time scales this complex diffusive behavior results in pseudo-Brownian motion wherein the mean-squared displacement (MSD) of diffusing molecules is proportional to time, but with a diffusion coefficient an order of magnitude smaller than that of Brownian motion^3^,^4^,^5^,^6^.

Corrals are 40 nm to 500 nm “picket fence” structures, recently confirmed to be comprised of plasma membrane-associated actin filament “fences” and transmembrane protein “pickets” anchored to the cytoskeleton^7^, which function to corral cytosolic and exofacial membrane elements respectively^3^,^5^,^6^,^7^,^8^. Additional diffusional heterogeneity on millisecond timescales arises through interactions between diffusing molecules and small dynamic domains such as lipid rafts and protein complexes. Although the biophysical properties of lipid rafts remain somewhat controversial, a generally accepted model of rafts has emerged, describing them as small (<50 nm), transient (a few ns to a few ms) assemblages of proteins, saturated lipids and cholesterol^9^,^10^,^11^. Clustering of raft elements, such as raft-borne receptors, stabilizes and enlarges rafts into longer-lived structures >100 nm in diameter^10^,^12^,^13^,^14^. Protein-protein interactions and the formation of protein microclusters also act to restrict diffusion in the plasma membrane^15^,^16^. These protein microclusters are not as well characterized as rafts, with the reported lifespans of these interactions ranging from milliseconds to hours^13^,^14^,^15^,^17^,^18^.

Interactions of diffusing lipids and proteins with corrals, rafts and other proteins result in emergent diffusional behavior that cannot be modeled as a Brownian process, but is better described by more complex models such as fractional Brownian motion or a subdiffusive continuous-time random walk^19^. While corrals, rafts and protein domains have been investigated individually, little is known of how they interact to produce the overall diffusional behavior observed in the plasma membrane. In this paper, we use a combination of moment scaling spectrum analysis (MSS), ensemble-based diffusional analyses, and caging analysis to assess the diffusional dynamics of the single-pass transmembrane protein CD93^20^,^21^,^22^. CD93 is a group XIV C-type lectin involved in angiogenesis, cell adhesion, inflammation, and the phagocytosis of apoptotic cells^23^,^24^,^25^,^26^, with most activity reported for a soluble form of the protein generated by MMP-mediated proteolysis^23^,^24^,^26^,^27^. Importantly for this study, CD93 appears to interact with only two cellular proteins, and therefore is expected to undergo relatively simple diffusion, free of any complexities that may arise when proteins diffuse as part of a large complex or engage in numerous intracellular interactions^28^,^29^. Our analyses of CD93 diffusion demonstrates the existence of previously undescribed, medium-lifespan, cholesterol-dependent membrane “cages” that are stabilized within corrals, and indicate the importance of these cages in restricting diffusion on spatial and temporal scales intermediate between those of rafts and corrals.

## Results

### CD93 Undergoes Anomalous Diffusion

**Figure 1:**
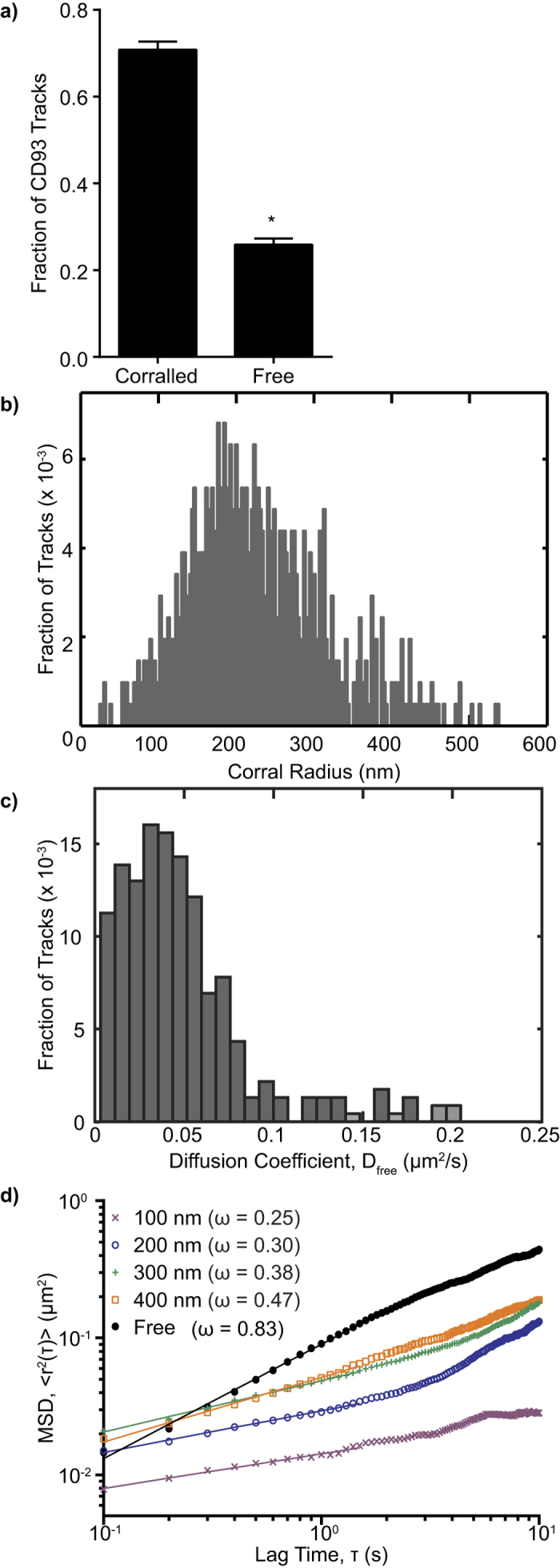
Diffusion of CD93 in Human Monocytes. (**a**) Classification of endogenous CD93 trajectories in monocytes as corralled or freely diffusing (free). (**b**) Distribution of CD93 diffusion corral radii. (**c**) Diffusion coefficient distribution of free (uncorraled) CD93. (**d**) Ensemble-averaged lag-time averaged MSD curves for CD93. The lines are fits to a power law over the range 0.1 ≤ τ ≤ 1.5 s, with the power law exponent, ω, for each line reported in the legend. ω = 1.0 corresponds to Brownian diffusion. n ≥ 5 independent experiments. (**b**–**d**) Data are plotted as means of pooled data from all experiments. *p < 0.05 compared to corralled, paired *t*-test.

CD93 diffusion was characterized using a combination of a robust single-particle tracking (SPT) algorithm and MSS analysis^20^,^21^. CD93 was labeled at low density using a full-length primary antibody and Cy3-monolabeled Fab fragments, a method which attaches single fluorophores to individual CD93 molecules without significant cross-linking^30^. MSS analysis^20^ classifies molecules as undergoing unconfined versus confined diffusion based on the relationship between CD93’s mean-displacement, mean-squared-displacement, mean-cubed-displacement and mean-quartic-displacement. The relationship between these quantities depends on whether the molecule’s diffusion is free (encounters no barriers to its movement), subdiffusive (is restrained in its movement compared to free diffusion, e.g. by the presence of a diffusional barrier) or super-diffusive (diffuses more rapidly than predicted of free diffusion, e.g. undergoes active transport). It is important to note that our measurements only detect motion which occurs over times longer than the 0.1 s interval between video frames, and as such CD93 classified as freely diffusive using this algorithm may be subdiffusive on shorter time scales. This could arise, for example, due to sub-millisecond interactions with other membrane components such as lipids^7^,^8^. Following MSS, SPT analysis then determines the diffusion coefficient of the free CD93 based on the initial slope of the MSD as a function of lag-time, and the size of confinement zones defined from the area explored by the confined molecules as defined by the 95^th^ percentile of CD93 step size. Applied to CD93 (Supplemental Video 1), these analyses identified distributions of corralled (confined) versus freely diffusing molecules, corral size, and free diffusion coefficients (Fig. 1a–c), with all of these values being consistent with those reported previously for other transmembrane proteins^4^,^31^,^32^,^33^,^34^. Moreover, the diffusion characteristics of CD93-GFP ectopically expressed on Chinese Hamster Ovary cells (CHO) and endogenous CD93 on monocytes were similar, and without any indication of significant CD93 oligomerization (Fig. 1, Supplemental Fig. 1e). The SPT analysis quantifies diffusion patterns using short lag times and assuming Brownian diffusion of both free and confined molecules; an assumption which is likely invalid in cellular membranes^19^,^21^. To address this limitation, and to extract more detailed information about the diffusion of CD93, we first subdivided our dataset based on the corral size determined from the SPT analysis, then calculated the ensemble-averaged lag-time-averaged MSDs^35^ for each subset (Fig. 1d, Supplemental Fig. 1f). This analysis revealed three distinct features. First, CD93 molecules identified as freely diffusing by SPT/MSS analysis were, in fact, subdiffusive over intermediate lag times (0.2 s < τ < 2 s). Secondly, CD93 molecules confined to smaller corrals were more subdiffusive than those in larger corrals. Finally, the logarithmic slope ω of the MSD plot increases at lag times >3 s, independent of corral size, indicating that CD93 becomes more diffusive as τ increases (Fig. 1d, Supplemental Fig. 1f).

### Anomalous Diffusion Occurs in Corrals

**Figure 2:**
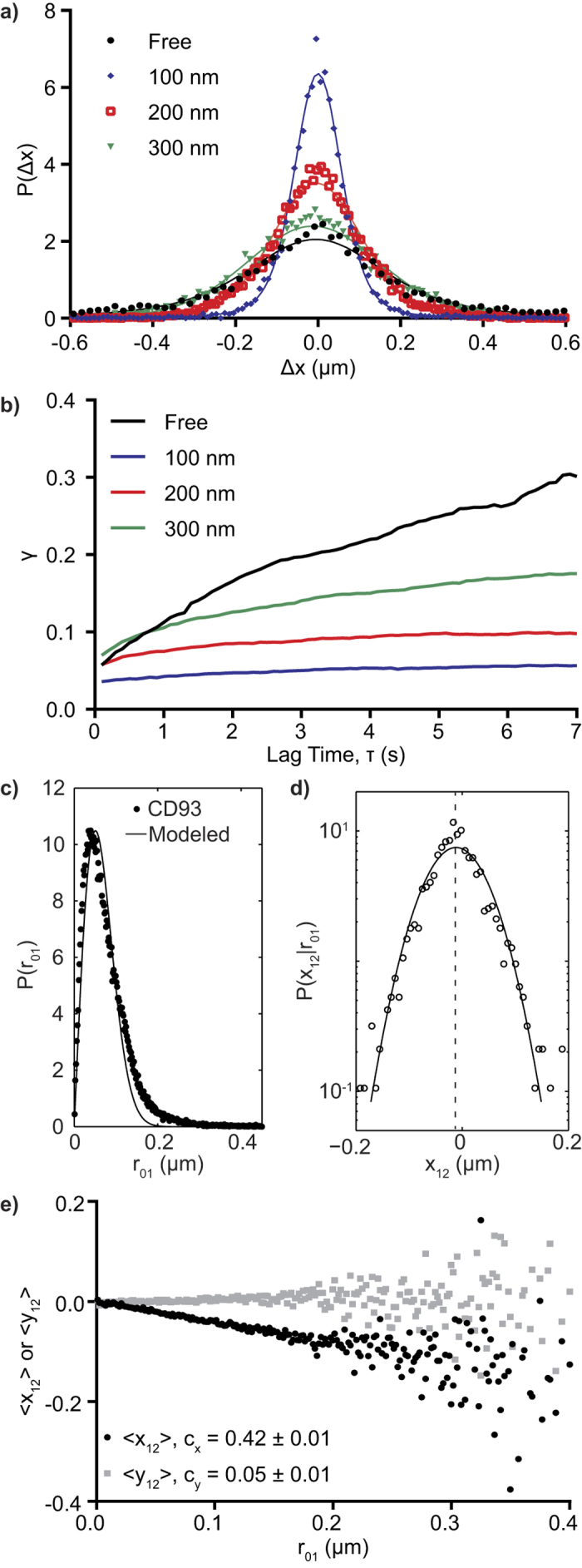
Characterization of Non-Brownian Diffusional Properties of CD93. Data are shown for CD93 identified by MSS analysis as undergoing free diffusion or confined to corrals of 100 nm, 200 nm and 300 nm. (**a**) Step-size probability distribution functions at a lag time of 1.5 s. (**b**) Evolution of molecular step-size as a function of lag time, as determined by the γ-scale parameter of an alpha-stable distribution fitted to the probability distribution for each population. (**c**) Probability distribution of first-step sizes compared to the distribution predicted for 2D Brownian diffusion with a mode of 40 nm (Modeled). (**d**) Normalized probability distribution *p*(*x*_12_|*r*_01_) as a function of *x*_12_ for *r*_01_ of 40 nm. A Gaussian fit to the data (solid line) gives 〈*x*_12_〉 = −0.01 (dashed line). (**e**) Mean parallel (〈*x*_12_〉) and perpendicular (〈*y*_12_〉) components of the second step as a function of first step magnitude *r*_01_. c_x_ and c_y_ are the negatives of the slopes of the 〈*x*_12_〉 and 〈*y*_12_〉, respectively, for *r*_01_ < 0.1 μm. n ≥ 5 independent experiments.

This behavior was further quantified by measuring the probability P(Δx) that a particle undergoes a displacement Δx over a given lag time. The shape (α-exponent) and width (γ-scale parameter) of P(Δx) were determined by fitting P(Δx) to an alpha stable distribution^36^. Brownian diffusion would give α = 2.0, with γ proportional to the diffusion coefficient. Figure 2a shows plots of P(Δx) for CD93 at a lag time of 1.5 s. Fits to an alpha-stable distribution give α < 2 in all cases, indicating that CD93 diffusion is non-Brownian (Table 1). As expected, the frequency of larger displacements increases with increasing lag time in freely diffusing populations, with γ increasing approximately linearly with τ (Fig. 2b). The freely diffusing population of CD93 was more likely to undergo larger displacements than corralled populations, with the maximum displacement in corralled populations being limited to the corral size. The γ-scale parameter evolved in corrals, increasing at short lag times and later plateauing (Fig. 2b).

To better understand the diffusion of CD93 within the corrals, we studied the correlation between the directions of successive particle steps using the cage analysis of Doliwa and Heuer^22^. On the time scale of our experiments, the freely diffusing molecules do not encounter any restrictions to their motions, and thus the directions of two successive steps will be uncorrelated. In contrast, molecules which move towards a diffusional barrier in one step are more likely to encounter that barrier and therefore move away from it in the next step, producing a negative correlation in the parallel component, but not the perpendicular component, of successive steps. This correlation is quantified by plotting the mean parallel component of the second step as a function of the size of the first step; from this plot the strength of the diffusion barrier is indicated by the magnitude of the plot’s slope, whereas the size of the diffusion barrier is defined by the first step size where this correlation breaks down^22^. A representative plot of the probability distribution of molecular steps of CD93 in a 100 nm corral (Fig. 2c) shows ~40 nm average step size and more frequent small steps than predicted for Brownian diffusion. The probability distribution of *x*_12_, the component of the second step parallel to the direction of a 40 nm first step *r*_01_, is Gaussian, with a non-zero negative mean 〈*x*_12_〉, consistent with caging of CD93 (Fig. 2d). This negative correlation was observed for all sizes of first steps, and the degree of correlation remained constant with increasing step size (Fig. 2e). In contrast, no significant correlation was observed in the perpendicular component 〈*y*_12_〉. Cage strength is indicated by c_x_, the negative of the slope of 〈*x*_12_〉 versus *r*_01_, while cage size is determined by the value of *r*_01_ at which this correlation breaks down^22^. For CD93 identified by SPT/MSS analysis as confined to 100 nm corrals, c_x_ = 0.42 ± 0.01, indicating the presence of a diffusional barrier. We did not, however, observe a breakdown of the correlation of 〈*x*_12_〉 versus *r*_01_, at first step sizes greater than the SPT-software-determined size of the corral (e.g. *r*_01_ > 100 nm, Fig. 2e). This lack of correlation between the size of cages and the size of the cage-containing corrals suggests that cages may not be the same structures as the corrals, and rather are separate structures of varying size found within corrals. Since the SPT/MSS and cage effect analyses use two conceptually distinct measures of confinement zone size, however, the size estimates obtained from these techniques may not be equivalent. It was therefore necessary to investigate other characteristics, such as lifetime, to determine whether cages and corrals are the same, or distinct, membrane structures.

### Molecular Cages are Dynamically Unique from Corrals

**Figure 3:**
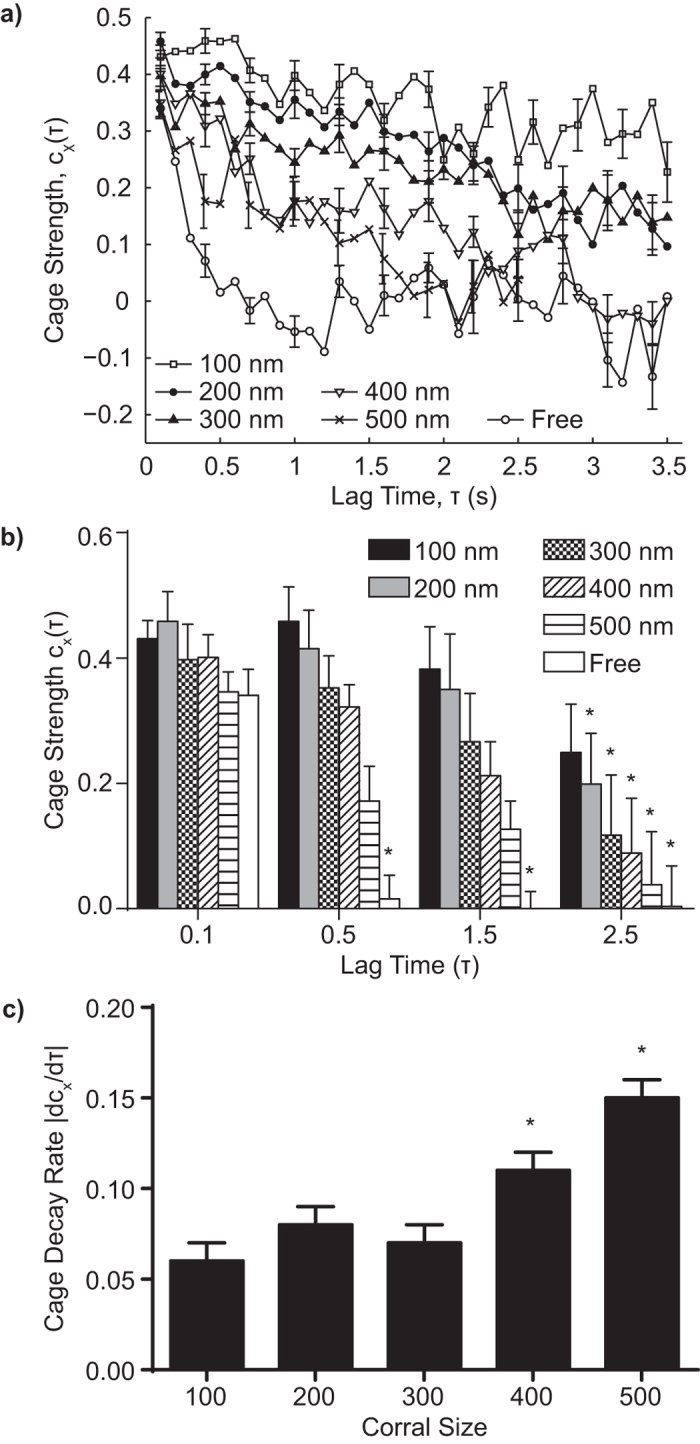
Cages are Dynamic Structures. Cage strength was quantified as the negative of the slope of 〈*x*_12_〉 with respect to *r*_01_. (**a**) Cage strength c_x_(τ) evolves as a function of lag-time for uncoralled CD93 (Free) and for CD93 in corrals. (**b**) Cage strength of uncorralled CD93 (Free) and CD93 in corrals 100 nm to 500 nm in size at selected lag times. (**c**) Cage decay rate. n = 5, *p < 0.05 compared to (**b**) cage strength of the same corral size at τ = 0.1 s, (**c**) 100 nm corals.

Previous studies have demonstrated that actin-based corrals are stable structures with lifetimes far longer than the 30 s duration of our experiments^4^,^6^. If the cages observed in Fig. 2 are the same cellular structures as corrals, we would expect the cage strength to remain constant for all lag times. In marked contrast, cage strength was observed to decrease as τ increased (Fig. 3a,b, Supplemental Fig. 2a,b), while the underlying corrals showed no decay (Supplemental Fig. 3), indicating that cages are shorter-lived structures than corrals. Interestingly, cage lifetime was inversely related to corral size, with cages in corrals of >300 nm decaying significantly faster than smaller cages (Fig. 3c, Supplemental Fig. 2c). Uncorralled CD93 also underwent caging, but with a much shorter half-life than corralled CD93 (Fig. 3b,c, Supplemental Fig. 2a,b), suggesting that the molecular processes that produce caging are ubiquitous throughout the membrane, but are stabilized by corrals in some fashion. Importantly, CD93 corralling and cages was identical on the apical and basolateral membranes (Supplemental Fig. 3d,e), demonstrating that both features are intrinsic to the cells and not due to interactions between the labeled CD93 and the underlying extracellular matrix or coverslip.

### Disruption of Actin or CD93 Function Does Not Alter Cage Strength or Lifespan

**Figure 4:**
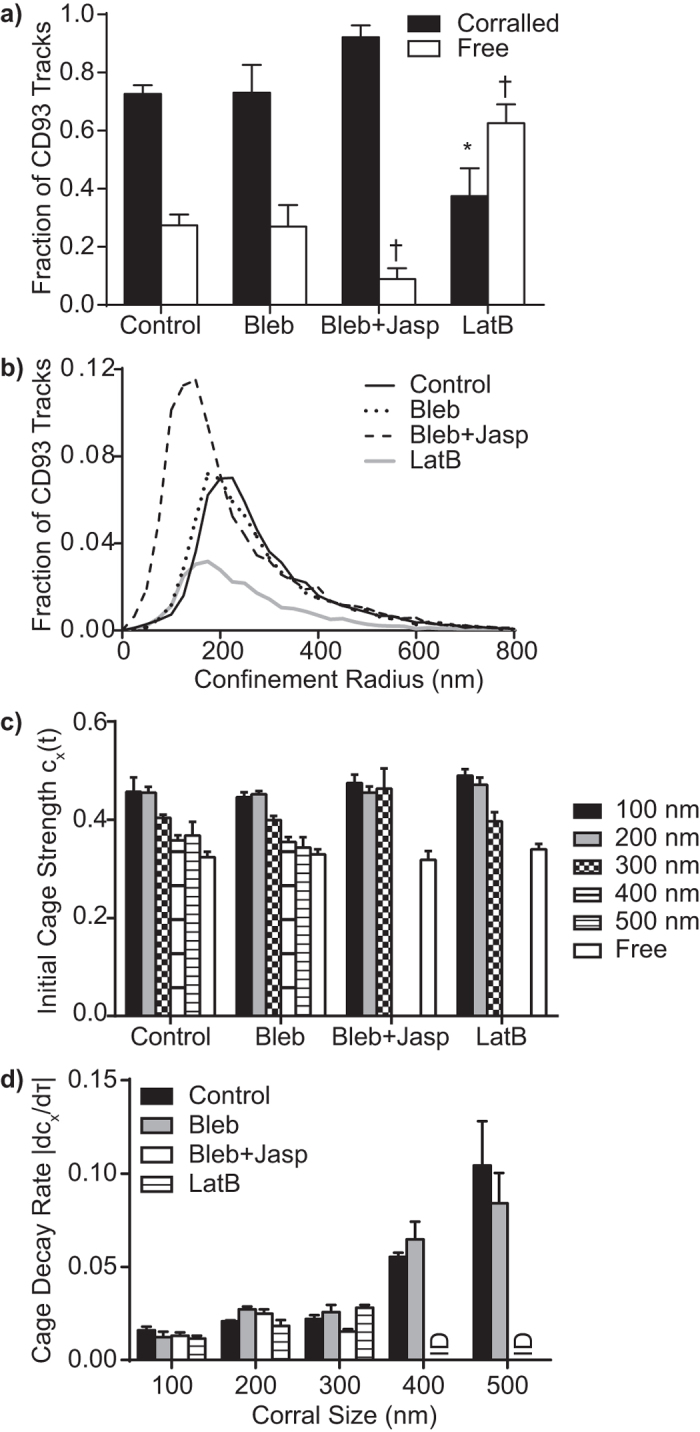
Cages are Structures Distinct from Actin-Based Corrals. The impact of actin stabilization (Jasplakinolide (Jasp) + Belbbistatin (Bleb)) and destabilization (Latrunculin B, LatB) were assessed on CD93 corralling and cage strength. (**a**) Effect of actin destabilization and stabilization on CD93 corralling. (**b**) Effect of actin stabilization and destabilization on the frequency and size of actin-based corrals, measured as fraction of total CD93 tracks. (**c**) Actin stabilization and destabilization do not affect cage strength at early lag times (τ = 0.1 s). (**d**) Rate of CD93 cage decay following stabilization or destabilization of the actin cytoskeleton. Data are expressed as mean ± SEM (**a,c,d**) or as mean (**b**) of 4 independent experiments. *^, †^p < 0.05 compared to fraction of Corralled (*) or Free (†) CD93 under control conditions (**a**), no statistical differences were observed (**c,d**). There was insufficient data to calculated cage strength and decay rates for 400 and 500 nm corrals treated with Bleb + Jasp or LatB (**c,d**).

To confirm that the cages were molecularly distinct structures from actin-based corrals, we employed actin-disrupting and stabilizing agents to manipulate corral stability. Latrunculin B-mediated actin destabilization and jasplakinolide-mediated actin stabilization were performed according to established protocols, with blebbistatin used in conjunction with jasplakinolide to prevent membrane blebbing^6^,^33^. As expected, stabilizing actin increased the portion of CD93 identified as corralled, whereas destabilization reduced CD93 confinement (Fig. 4a) and destabilized the remaining corrals such that a significant portion of corralled CD93 escaped from the remaining corrals over the course of the experiment (Supplemental Fig. 3). Both stabilization and destabilization decreased average corral size but had opposing effects on corral frequency (Fig. 4b), likely due to increases in cortical actin density and stability following jasplakinolide treatment and preferential disruption of larger corrals by Latrunculin B. Despite the profound effects of actin stabilization and destabilization on CD93 corrals, these manipulations had no impact on either cage strength (Fig. 4c) or cage lifetime (Fig. 4d), confirming that while stabilized by actin-derived corrals, these cages are not themselves actin-dependent structures.

Cages may also be a property of CD9’3 biological activity. Indeed, CD93 is a C-type lectin which can act as an adhesion molecule, and furthermore, CD93 can be directly cross-linked to the cytoskeleton through interactions with moesin^25^,^29^. To determine if these interactions were responsible for forming cages, we performed cage-effect analysis on ectopically expressed deletion mutants lacking either the C-type lectin domain or intracellular domain of CD93, and observed no significant impact on the corralling (Supplemental Fig. 4a,b), nor on the initial cage strength or the rate of cage decay (Supplemental Fig. 4c,d), demonstrating that caging and corralling are independent of CD9’3 functional domains.

### Cholesterol Enhances Cage Strength and Stability

**Figure 5:**
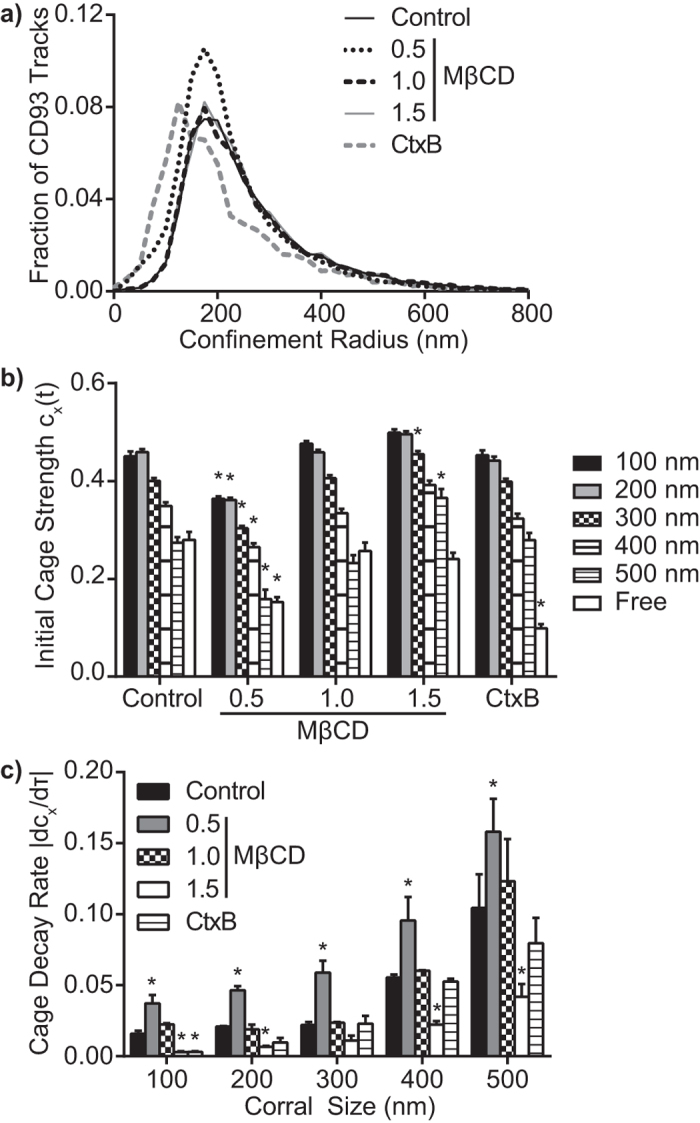
Cholesterol Stabilized Cages. The effect of altered plasma membrane cholesterol content and CtxB-mediated raft stabilization was quantified in terms of (**a**) corral size, (**b**) initial cage strength (τ = 0.1 s), and (**c**) cage decay rates. Data are plotted as mean (**a**) or mean ± SEM (**b,c**) of 3 independent experiments. For MβCD, 0.5, 1.0 and 1.5 indicate the quantity of membrane cholesterol relative to control cells following MβCD treatment. *p < 0.05 compared to the same corral size under control conditions.

The independence of cage stability and strength from actin stability suggests that the cages are not made of actin. Cholesterol-stabilized domains such as lipid rafts are a possible class of structures that could account for the observed properties of cages^37^. To test this, we utilized well-established protocols to modify the abundance of cholesterol in CHO cells^38^. Depletion of cholesterol is known to reduce raft stability, while increasing cholesterol content stabilizes rafts^39^. We found that depleting cholesterol increased the fraction of CD93 which was corralled, but not the corral size. Other manipulations of cholesterol content had no significant effect on the fraction of corralled CD93 or on corral size (Fig. 5a, Supplemental Fig. 5a), and only cholesterol depletion had an impact on the diffusion coefficient of CD93 (Supplemental Fig. 5b). Cholesterol depletion decreased initial cage strength in corrals of all sizes, whereas increasing cholesterol concentration tended to increase initial cage strength (Fig. 5b). Depletion of plasma membrane cholesterol levels also destabilized cages, reducing their lifetime by roughly a factor of two (Fig. 5c). Although measurements of cage strength and lifespan are independent of the diffusion coefficient, and our manipulation of cholesterol had no discernable effect on CD93 diffusion or cell morphology (Fig. 5, Supplemental Fig. 5c), cholesterol depletion or supplementation may have had impacts on interactions between membrane constituents that are independent of membrane fluidity^22^,^40^,^41^,^42^. To address this issue we used raft-stabilizing cholera toxin subunit B (CtxB) to assess the role of cholesterol-stabilized domains without altering membrane fluidity or cholesterol content. CtxB is a pentameric protein which cross-links the ganglioside GM1, a lipid raft component, thereby producing stable cholesterol-enriched rafts ~100 nm in diameter^10^. CtxB had no significant effect on the corralling or diffusion of CD93 (Fig. 5a, Supplemental Fig. 5a,b), but dramatically stabilized small cages, selectively increasing the lifetime of 100 nm cages by a factor of 5 (Fig. 5c).

## Discussion

We have demonstrated the existence of a membrane compartment which is stabilized within membrane corrals and by cholesterol. These domains, which we have termed “cages”, act to restrict protein diffusion over periods of hundreds of milliseconds up to a few seconds. Importantly, these cages form independently of the underlying actin cytoskeleton or functional domains in CD93, indicating that they are likely a general membrane structure rather than a product of CD93 biology. While domains with similar diffusion-restricting properties have been created in model membranes containing a small number of lipid species and a single transmembrane protein^43^, to our knowledge this is the first report of cage-like domains, and of their dependence on actin and cholesterol for stability, in the plasma membrane of living cells. While the biological role of these structures remains unelucidated, the stabilization of cages in areas within actin-based membrane corrals and their raft-like dependence on cholesterol suggests that their function may share aspects with those of both corrals and rafts. Diffusional barriers such as corrals function predominantly through receptor immobilization and lateral structuring of signaling components. For example, FcεR1 signaling is enhanced by corrals through acceleration of FcεR1 immobilization following ligation^6^. Corrals serve two critical functions in FcγR signaling – they prevent the spontaneous aggregation of unligated receptors, and exclude inhibitory phosphatases from sites of active signaling^15^,^34^. In contrast to corrals, lipid rafts are small, transient structures comprised of cholesterol-stabilized islands of phospholipids, sphingolipids and proteins^10^,^40^,^44^,^45^,^46^. Lipid rafts are more dynamic than corrals, with lifetimes ranging from a few tens of ms under resting conditions, to several seconds following receptor crosslinking. Stabilized rafts function to selectively scaffold proteins and lipids into functional signaling complexes, but have a much smaller effect on restricting diffusion^13^,^14^,^47^,^48^,^49^. Like rafts, cages require cholesterol for stability, while like corrals, they act to restrict protein mobility in the plasma membrane, suggesting that cages may represent a longer-lived lipid-raft like domain which structures membrane components with the added function of confining diffusion.

The preferential stabilization of cages within corrals (Fig. 3) and the concomitant dependence of initial raft strength and lifespan on cholesterol may be explained by the presence of transmembrane “pickets” which line the edges of corrals. Transmembrane proteins exclude cholesterol from the annular ring of lipids surrounding the transmembrane domain, as the irregular surface of the helical transmembrane domain does not pack efficiently with the relatively short and linear structure of cholesterol^50^,^51^. A portion of the excluded cholesterol is likely concentrated within the corral^52^, where it would contribute to the formation and stability of cages. This local increase in cholesterol would also explain the inverse relationship between corral size and cage stability (Fig. 3) as the ratio of the volume of the bounding torus of picket proteins to the volume of the enclosed corral scales inversely with corral diameter. For example, a 100 nm diameter circular corral with transmembrane pickets 3 nm in diameter would have a picket volume ~12% that of the enclosed corral; this drops to 6% for a 200 nm corral and 2% for a 500 nm corral. Smaller corrals would thus experience higher relative increases in cholesterol, accounting for the observed independence of cage stability on corral size.

It is tempting to speculate that cages are simply rafts stabilized by increased cholesterol concentration within corrals. While there is a great deal of controversy surrounding the size, lifespan, and even existence of rafts (reviewed in refs 11,53 and 54), the growing consensus is that rafts are nanometer-sized, transient structures, rather than larger, stable membrane structures. This refinement of accepted raft size and stability is a product of new methodologies with improved temporal and spatial resolution compared to historical biochemical and fluorescent imaging methods. Direct measurements of the sphingolipid component of rafts by STED–fluorescence correlation spectroscopy measured raft lifespans two to three orders of magnitude shorter, and raft sizes an order of magnitude smaller, than the lifespans and sizes of the cages observed here (10–20 ms, <20 nm)^9^. Similarly sized (6–20 nm), but shorter lived (0.5–1.0 ms) rafts were observed using ultra-high-speed single-particle tracking of lipids in model membranes^37^. Measurements of both GPI-linked and transmembrane-domain-containing raft-born proteins show similar trends, although the stability and size of protein-bearing rafts tend to be larger than that observed for raft lipid components (100–530 ms, 40–250 nm)^13^,^55^,^56^,^57^. Even given the broad range of potential raft lifetimes and sizes, the cages observed in the present study have lifespans several orders of magnitude longer, and sizes several times larger, than the largest values reported for rafts on unstimulated cells. In addition, the trend in stability we observe for cages runs counter to that observed for rafts, with the reported lifespans of lipid rafts trending towards longer lifespans with increasing raft size^13^,^55^,^56^,^57^,^58^,^59^, whereas we observed a decrease in cage lifetimes as cage size increased (Fig. 3C). Moreover, CD93 lacks the usual markers of a protein which is recruited to lipid rafts, such as palmitoylation, nor is it known to interact with any raft-bound proteins, and therefore would not be expected to localize to rafts^28^,^60^,^61^. Combined, these results indicate that cages are structures distinct from rafts.

The lifespan and size of our cages appears to be more similar to those of protein-based microdomains^62^. Of the many protein-based microdomains and large membrane complexes that have been reported in the literature (e.g. focal contacts, caveoli), the cages observed in this study are most consistent with the properties of tetraspanin-enriched microdomains^63^,^64^,^65^. As with our cages, tetraspanin-enriched microdomains are stabilized by cholesterol but remain structurally distinct from lipid rafts^62^,^66^, have a size distribution similar to what we have observed for cages^31^,^62^,^67^, and can limit the diffusion of other transmembrane proteins^62^,^63^,^68^. Furthermore, some tetraspanins can directly interact with actin^68^,^69^, suggesting that corrals may enhance cage stability through the formation of stable actin-tetraspanin complexes rather than by concentrating cholesterol. While we have not been able to co-immunoprecipitate CD93 with any tetraspanin or other transmembrane or membrane-associated proteins (data not shown), it is possible that the transient nature of cages may preclude efficient capture of cage components through conventional biochemical techniques. While the characteristics of tetraspanins are consistent with those of cages, other multiprotein complexes are also potential cage candidates. Indeed, many protein complexes in cell membranes can be disrupted by cholesterol depletion^16^, consistent with the destabilizing effect of cholesterol depletion on cage stability (Fig. 5). However, caging is likely not a general feature of membrane protein complexes, as many protein complexes are disrupted by cholesterol supplementation^16^, whereas supplementation enhanced cage stability (Fig. 5). If these caging interactions occur strictly through protein-protein interactions, then the strength of these interactions is likely weak, as most reported protein-protein interactions restrict diffusion over time periods several orders of magnitude larger than the observed lifespan of cages^70^,^71^,^72^,^73^. Taken together, our results demonstrate the presence of a cholesterol-dependent diffusional compartment which forms throughout the plasma membrane and is stabilized by actin corrals. While the complete molecular composition of this domain remains to be elucidated, cages share several characteristics with both lipid-based and protein-based microdomains, and act to limit diffusion on temporal and spatial scales intermediate between raft-like domains and actin-dependent corrals.

## Materials and Methods

### Materials

Lympholyte-poly and Cy3-conjugated rabbit-anti-mouse Fab fragments were purchased from Cedarlane. Ham’s F12, DMEM, 100X antibiotic-antimycotic solution and fetal bovine serum (FBS) were purchased from Wisent. #1.5 thickness, 18 mm round coverslips were purchased from Electron Microscopy Supplies. Anti-human CD93 (clone R139) was purchased from eBioscience. Leiden chambers were purchased from Quorum instruments. Latrunculin B, methyl-β-cyclodextran, blebbistatin and jasplakinolide were purchased from Cayman chemical. Cholesterol was purchased from Advanti Polar Lipids. GenJet Plus *In Vitro* DNA Transfection Reagent was purchased from SignaGen Laboratories. The Leica DMI6000B microscope and all components were purchased from Leica Microsystems. Matlab equipped with the parallel processing, statistics, image processing, and optimization toolboxes was purchased from Mathworks. All other materials were purchased from Thermo Fisher.

### Human Monocyte Isolation

The collection of blood from healthy donors and all subsequent experiments were approved by the Health Science Research Ethics Board of the University of Western Ontario. Venipuncture was performed in accordance with the guidelines of the Tri-Council Policy Statement on human research. All subjects provided informed consent prior to venipuncture. Blood was drawn into heparinized vacutainer collection tubes, layered over an equal volume of Lympholyte-poly cell separation media and centrifuged at 300 × g for 35 min at 20 °C. The top band of peripheral blood mononuclear cells was collected, washed once (300 × g, 6 min, 20 °C) with phosphate-buffered saline (PBS, 137 mM NaCl, 2.7 mM KCl, 10 mM Na_2_HPO_4_, 1.8 mM KH_2_PO_4_), and the cell pellet suspended in RPMI-1640 + 10% FBS and 1% antibiotic-antimycotic solution at a density of ~2 × 10^6^ cells/ml. Monocytes were isolated from this mixture by incubating 250 μl of the cells on sterile 18 mm #1.5 thickness coverslips for 1 hr at 37 °C, followed by removal of non-adherent cells by gentle washing with RPMI 1640 + 10% FBS. Cells were maintained at 37 °C/5% CO_2_ for a maximum of 6 hours prior to use.

### CHO Cell Culture and Transfection

CHO cells were cultured in DMEM or F-12 media + 10% FBS in a 37 °C/5% CO_2_ incubator. The cells were split 1:10 upon reaching 80% of confluency by washing once with PBS, followed by a 2 min incubation in 0.25% Trypsin-EDTA. For imaging experiments, the cells were cultured on sterile 18 mm round coverslips housed in 12-well plates. For each coverslip, 1 μg of human CD93-GFP and 2.25 μl of GenJet reagent were individually suspended into 38 μl of serum-free DMEM. The two suspensions were then mixed, incubated for 10 min at 20 °C, and then added drop-wise to the cells. Cells were used ~24 hrs after transfection.

### Generation of C-type Lectin and Intracellular Domain CD93 Deletion Mutants

PCR mutagenesis was used to delete the C-type lectin domain (ΔCTLD) and intracellular domains (ΔID) from CD93-GFP. PCR was performed using Phusion DNA polymerase as per the manufacturer’s instructions, using 5′ phosphorylated primers flanking amino acids 32–174 (CTLD-FWD: GCAGA CCACC GCCTC CGTGT, CTLD-REV: ATTGA GGGCT TCGTG TGCAA GT) or flanking amino acids 602–652 (ID-FWD: CAGAG CCAGG GCCAG CAGGA GTAG, ID-REV: ACCGG TCGCC ACCAT GGTGA), 35 cycles of 30 sec annealing at 61 °C and 7 min elongation at 72 °C. The parental plasmid was removed by addition of 1:50 v/v DpnI followed by incubation for 2 hrs at 37 °C. The PCR product was then gel purified, recirculized by incubating with T4 DNA ligase overnight at 16C, transformed into chemically competent DH5α *E. coli*, and recovered on LB-kanamycin plates. Deletions were confirmed by Sanger sequencing (London Regional Genomics Centre, London, Canada).

### Immunolabeling of CD93

CD93 was immunolabled using standardized protocols for single particle tracking, using a combination of low antibody concentration and Fab secondary antibodies to label CD93 without crosslinking and at a sufficiently low label density to allow resolution of individual point-spread functions^6^,^30^,^31^,^71^. Briefly, monocytes or transfected CHO cells were cooled to 10 °C and blocked for 20 min in F12 media + 4% human serum, then incubated for 20 min with blocking solution containing 1:5,000 anti-human CD93. The cells were then washed 3 × 5 min in PBS and incubated with blocking buffer containing 1:1000 dilution of Cy3-conjugated rabbit-anti-mouse Fab for an additional 20 min. The samples were washed an additional 3 × 5 min in F12 media + 10% FBS and kept at 10 °C until imaged.

### Disruption of the Actin Cytoskeleton

Actin-based corrals were disrupted and stabilized using the protocol of Flannagan *et al*.^33^. In brief, CHO cells were transfected with GFP-CD93, labeled with anti-CD93, and control single particle videos recorded as described below. For corral disassembly experiments, the media in the Leiden chamber was replaced with 4 μM latrunculin B and incubated for 20 min at 37 °C before re-imaging the sample. For corral stabilization experiments the sample was re-imaged twice; first following a 20 min/37 °C incubation with 60 μM blebbistatin, and again following a 10 min/37 °C incubation with 60 μM blebbistatin plus 1 μM jasplakinolide. A minimum of 20 cells were recorded per treatment on each coverslip.

### Cholesterol Depletion/Enrichment and Raft Stabilization

Plasma membrane cholesterol levels of CHO cells were modulated using the method of Romanenko *et al*.^38^. Cholesterol-saturated Methyl-β-cyclodexran (MβCD) was prepared by drying 100 μl of a 20 mg/ml solution of cholesterol in 1:1 chloroform-methanol under nitrogen. After drying, 1 ml of 5 mM MβCD in serum-free Ham’s F-12 medium was added, then the tube was vortexed and incubated overnight in a 37 °C/1500 RPM shaking heat-block. The resulting solution of cholesterol-saturated MβCD was then mixed with a 5 mM solution of cholesterol-free MβCD at ratios of 0:1, 4:1 and 9:1, to alter cellular cholesterol levels to 0.5×, 1.0× and 1.5× of untreated cells, respectively^38^. CD93-GFP transfected CHO cells were then washed 3× with serum-free F-12 media and incubated with the cholesterol-MβCD mixtures for 120 min at 37 °C/5% CO_2_. After incubation the cells were washed 3× with serum-free media, labeled with anti-CD93, and a minimum of 5 single particle videos recorded per coverslip as described above. CtxB-mediated raft stabilization was performed using the method of van Zanten *et al*.^10^. Briefly, CD93-GFP transfected CHO cells were labeled with the primary anti-CD93 antibody as described above, but with 10 μg/ml unlabeled CtxB added along with the secondary Cy3-labeled Fab. Cells were then washed 3× with serum-free media, and a minimum of 20 single particle videos recorded per coverslip.

### CD93 Single Particle Tracking

Single particle tracking of CD93 was performed on a Leica DMI6000B microscope equipped with a 100×/1.40NA objective, supplemental 1.6× in-line magnification, a photometrics Evolve-512 delta EM-CCD camera mounted using a 0.7× adaptor (total system magnification of 112×), a Chroma Sedat Quad filter set and a heated/CO_2_ perfused live-cell piezoelectric stage. Coverslips were placed into a Leiden chamber containing 1 ml of imaging buffer (150 mM NaCl, 5 mM KCl, 1 mM MgCl_2_, 100 μM EGTA, 2 mM CaCl_2_, 25 mM HEPES and 1500 g/L NaHCO_3_) pre-warmed to 37 °C. The Leiden chamber was then mounted on the microscope’s live-cell stage and maintained at 37 °C/5% CO_2_ for the duration of the experiment. The microscope was focused on the basolateral side of the cells which is in contact with the coverslip, and 30 sec duration videos were recorded using the Cy3 (Ex: 550 ± 25 nm, Em: 605 ± 52 nm) channel at a magnification of 113x and a frame rate of 10 frames per second. 5 to 20 cells were recorded on each coverslip. The resulting videos were cropped to a minimum bounding area containing the cell under observation and exported as TIFF stacks. SPT was performed using the Matlab scripts of Jaqaman *et al*.^21^, and custom-written Matlab scripts used to remove any CD93 trajectories with a positional accuracy worse than 25 nm, with a median positional accuracy in most experiments better than 15 nm. Only datasets with >1,000 trajectories following filtering were used for subsequent analysis. An initial characterization of CD93 diffusion was performed for all datasets using the SPT and MSS analysis scripts included in the software of Jaqaman *et al*.^21^ Briefly, the MSS analysis^20^ classifies molecules as undergoing unconfined versus confined diffusion based on the power-law indices, γ_p_, of the first through fourth moments, 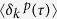, of molecular movement, where each moment is assumed to have a power-law dependence on the lag time, with 
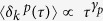
. The relationship between p and y_p_ provides information about the mode of diffusion, identifying it as Brownian, subdiffusive (confined) or superdiffusive. SPT analysis then quantifies the diffusion coefficient of free CD93 based on the initial slope of the MSD plot. For confined CD93, the confinement zone size is defined as the 90^th^ percentile of the maximum possible extent of the corral, as quantified by the variance-covariance matrix of the CD93 molecules’ trajectories^21^. Oligomerization of CD93 was quantified by measuring the amplitude of individual diffusion-limited PSFs in each image, and normalizing the amplitude to the modal amplitude of the data set^21^,^31^.

### Ensemble-averaged Time-averaged MSD Curves and Probability Distribution Functions

To compare the diffusion behavior of CD93 molecules from different sub-populations or cell types, the ensemble-averaged time-averaged MSD, defined as


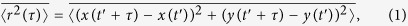


was calculated for each group for lag times 0.1 ≤ τ ≤ 10 s. These MSDs were determined, and all other calculations performed, using custom-written Matlab scripts unless otherwise stated.

Particle displacements in the *x* and *y* directions were extracted from the CD93 trajectories for each desired lag time and population. Histograms of these displacements served as approximations to the normalized probability density functions (PDF). Each PDF was fitted to a Gaussian distribution as well as to a family of alpha-stable distributions using a third-party Matlab toolbox^36^. PDFs for the magnitude of the 2D particle displacements were also fitted to the distribution expected for Brownian diffusion,


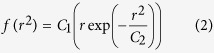


where *C*_1_ and *C*_2_ were treated as fitting parameters.

### Cage Effect Analysis

Cage effect analysis was performed as per the algorithm developed by Doliwa and Heuer^22^. In brief, for each lag time τ and each CD93 molecule in an ensemble, the positions of the CD93 molecule at three successive time points, 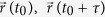 and 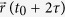, were used to calculate the vectoral movement of two successive steps made by the molecule, 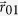 and 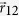. The parallel (*x*_12_) and perpendicular (*y*_12_) components of the second step relative to the direction, expressed as the unit vector 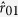, of the first were then determined, with 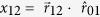 and 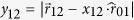. This procedure was performed for all CD93 molecules in the ensemble at all values of *t*_0_. The conditional probability distribution *p*(*x*_12_|*r*_01_) of a CD93 molecule moving a parallel distance *x*_12_ on its second step, given a first step length of *r*_01_ was constructed, as was the analogous probability distribution *p*(*y*_12_|*r*_01_) for the perpendicular component. Caging was indicated by a negative correlation between 〈*x*_12_(*r*_01_)〉, the mean of *p*(*x*_12_|*r*_01_), and *r*_01_. Cage strength is characterized by the negative of the slope of 〈*x*_12_(*r*_01_)〉 versus *r*_01_. No caging was observed when cage effect analysis was applied to monodispersed stationary fluorophores, indicating that caging is a product of biological restriction of diffusion and not due to noise-induced errors in the precision of CD93 localization (data not shown).

### Statistics

Statistical analyses were performed in Graphpad Prism 6 or using Mathwork’s Matlab. ANOVA with Tukey multiple corrections test was used for all analyses unless otherwise noted.

## Additional Information

**How to cite this article**: Goiko, M. *et al*. Short-Lived Cages Restrict Protein Diffusion in the Plasma Membrane. *Sci. Rep.* **6**, 34987; doi: 10.1038/srep34987 (2016).

## Supplementary Material

Supplementary Video 1

Supplementary Information

## Figures and Tables

**Table 1:** α and γ fit values determined from fits of P(Δx) for CD93 to alpha-stable distributions at a lag time of 1.5 s.

Population*	Monocyte		CHO	
	α	γ	α	γ
Free	1.44 ± 0.06	0.141 ± 0.007	1.39 ± 0.02	0.153 ± 0.003
100 nm	1.62 ± 0.07	0.045 ± 0.002	1.58 ± 0.01	0.049 ± 0.001
200 nm	1.77 ± 0.04	0.081 ± 0.003	1.81 ± 0.02	0.084 ± 0.001
300 nm	1.85 ± 0.03	0.118 ± 0.004	1.82 ± 0.03	0.121 ± 0.002
Brownian†	2.00	ǂ	2.00	ǂ

^*^As determined by MSS analysis^20^.

^†^Theoretical values for a Brownian population.

^ǂ^Proportional to diffusion coefficient.
