# Supplementary material for: Short-Lived Cages Restrict Protein Diffusion in the Plasma Membrane
Source: Sci Rep. 2016 Oct 11;6:34987. doi: 10.1038/srep34987 (PMC5057110; doi:10.1038/srep34987)
Supplement: Supplementary Information [file srep34987-s2.pdf]

Short-Lived Cages Restrict Protein Diffusion in the Plasma Membrane

Maria Goiko, John R. de Bruyn, Bryan Heit.

Supplemental Figure Legends

Supplemental Figure 1: Diffusion of CD93-GFP in CHO cells. a) Montage of CD93-GFP trajectories in CHO cells. Tracks were defined as free, confined or directed by Moment Scaling Spectrum analysis, with confined tracks further subdivided into ensembles with mean confinement radii of 50-150 nm (100 nm), 150-250 nm (200 nm), or 250-350 nm (300 nm). **b)** Classification of ectopically expressed CD93-GFP trajectories in CHO cells as corralled or freely diffusing (free). **c)** Distribution of CD93-GFP corral radii. **d)** Diffusion rate distribution of free (uncorralled) CD93-GFP. **e)** CD93 oligomerization of endogenous CD93 on human monocytes (WT) versus ectopically expressed CD93-GFP on CHO cells (GFP). Oligomerization is quantified as the amplitude of the point-spread function of individual diffusion-limited spots. **f)** Ensemble-averaged lag-time averaged MSD curves for CD93-GFP. The lines are fits of data in the range $0.1 \leq \tau \leq 1.5$ s to a power law with exponent α, where $\alpha = 1.0$ indicates Brownian diffusion. $n \geq 5$ independent experiments. C-E, Data are plotted as mean of pooled data from all experiments. * $p < 0.05$ compared to Corralled, paired *t*-test.

Supplemental Figure 2: Cages are Dynamic Structures. Cage strength was characterized in CD93-GFP expressing CHO cells as the negative of the slope of a plot of $\langle x_{12} \rangle$vs. r_{01}. **a)** Cage strength evolves as a function of lag time for uncorralled CD93 (Free) and CD93 in corrals 100 nm to 500 nm in size. **b)** Cage strength, $c_x(\tau)$, at selected lag times for uncorralled CD93 (Free) and CD93 in corrals 100 nm to 500 nm in

size. **c)** Rate of cage decay. **d)** Fraction of corralled versus freely diffusing CD93-GFP on the apical versus basolateral sides of CHO cells. **e)** Initial cage strength of CD93-GFP cages on the apical versus basolateral sides of CHO cells. n = 5 (a-c) or 2 (d-e), * $p < 0.05$ compared to (b) cage strength of the same corral size at $\tau = 0.1$ s, (c) 100 nm corals.

Supplemental Figure 3: Actin Destabilization Weakens Corrals. Power-law indices were calculated for CD93 confined in 200 nm corrals from Time-Averaged MSDs from vehicle-treated CD93-GFP expressing CHO cells (Control), or in CHO cells treated with Blebbistatin (Bleb), Blebbistatin + Jasplakinolide (Bleb+Jasp) or with Latrunculin B (LatB). **a)** Distribution of power-law indices after 2 sec of experimental time. **b)** Distribution of power-law indices after 8 sec of experimental time; Latrunculin B treated cells display a secondary population with a mean $\alpha \approx 1.0$, indicating a portion of this CD93 has escaped the corral and is freely diffusing. Data is displayed as the ensemble data of 3 independent experiments.

Supplemental Figure 4: CD93 Diffusion is not Regulate by CTLD or Intracellular Domains. Corralling and Caging of wild-type CD93 (WT) versus CD93 lacking either the C-Type Lectin Domain (ΔCTLD) or Intracellular Domain (ΔID) in CHO cells. **a)** Fraction of diffusing CD93 which is corralled versus freely diffusing. **b)** Distribution of CD93 corral sizes. **c)** Initial cage strength ($\tau = 0.1$ s) for uncorralled CD93 (Free) and CD93 in corrals 100 nm to 500 nm in size. **d)** Cage decay rate of corralled CD93 in corrals 100 nm to 500 nm in size. n = 4, no statistically significant differences were observed, ANOVA with Tukey correction.

Supplemental Figure 5: Membrane Cholesterol Content Regulates CD93 Diffusion. The effect of altered plasma membrane cholesterol content and CtxB-mediated raft stabilization in CD93-GFP expressing CHO

cells was quantified in terms of **a)** the proportion of CD93 undergoing confined versus free diffusion, and **b)** the diffusion coefficient of free (uncorralled) CD93. **c)** Distribution of CD93-GFP and cell morphology of CHO cells after manipulation of plasma membrane cholesterol levels. Images are representative of 3 independent experiments, scale bars are 10 μm. *, † = $p < 0.05$ compared to Corralled (*) or Free (†) under control conditions (a), * = $p < 0.05$ compared to control (b). For MβCD, 0.5, 1.0 and 1.5 indicate the quantity of membrane cholesterol relative to control cells following MβCD treatment, CtxB indicates cholera toxin subunit B treatment.

Supplemental Video 1: Representative CD93 Single-Particle Tracking Video. Representative video of immunolabeled CD93-GFP tracked on the basolateral surface of a transfected CHO cell. Left panel shows the raw video; right panel shows an overlay of CD93 trajectories over the video. Green tracks indicate freely diffusing CD93, red tracks indicate confined CD93. Scale bar is 10 μm, each frame is 0.1 s in duration.

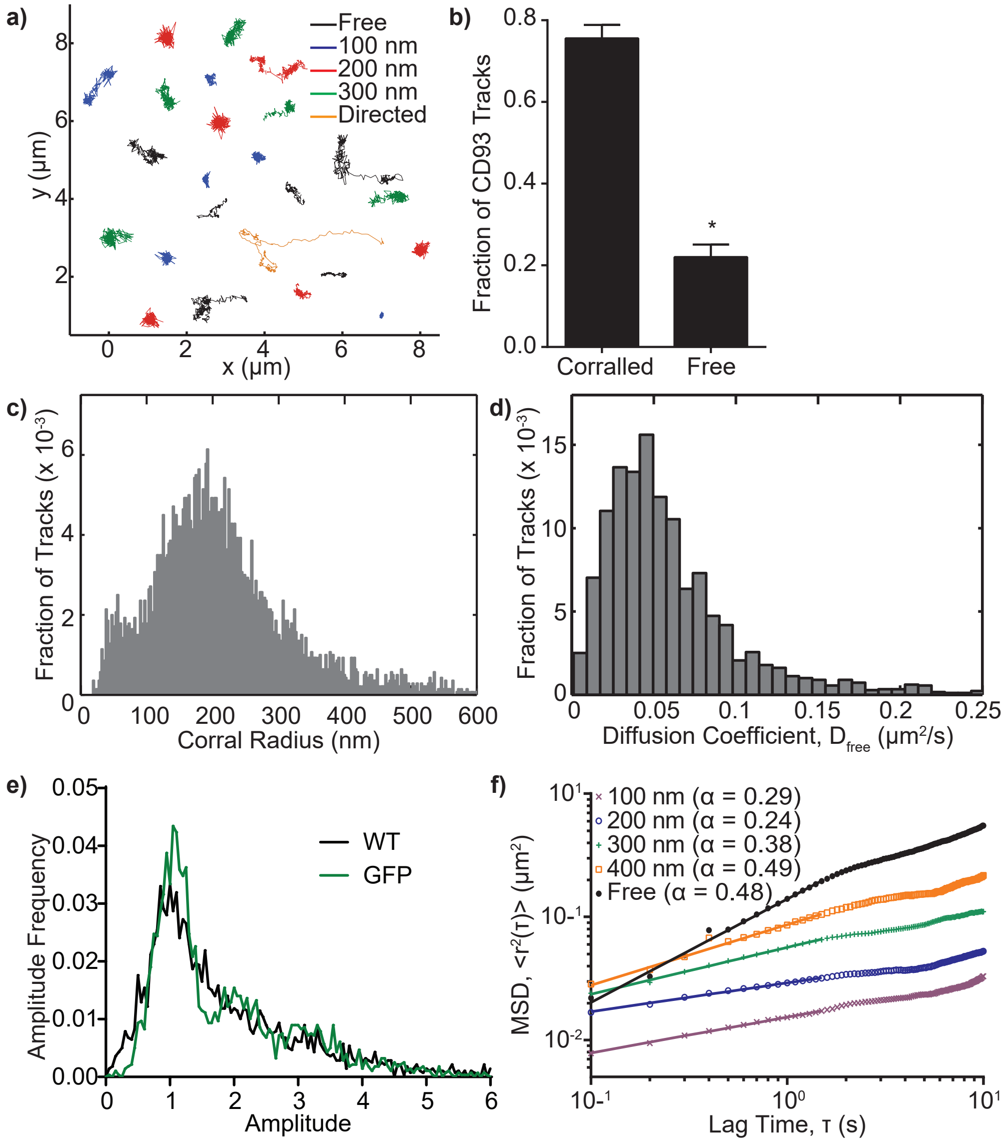

Supplemental Figure 1

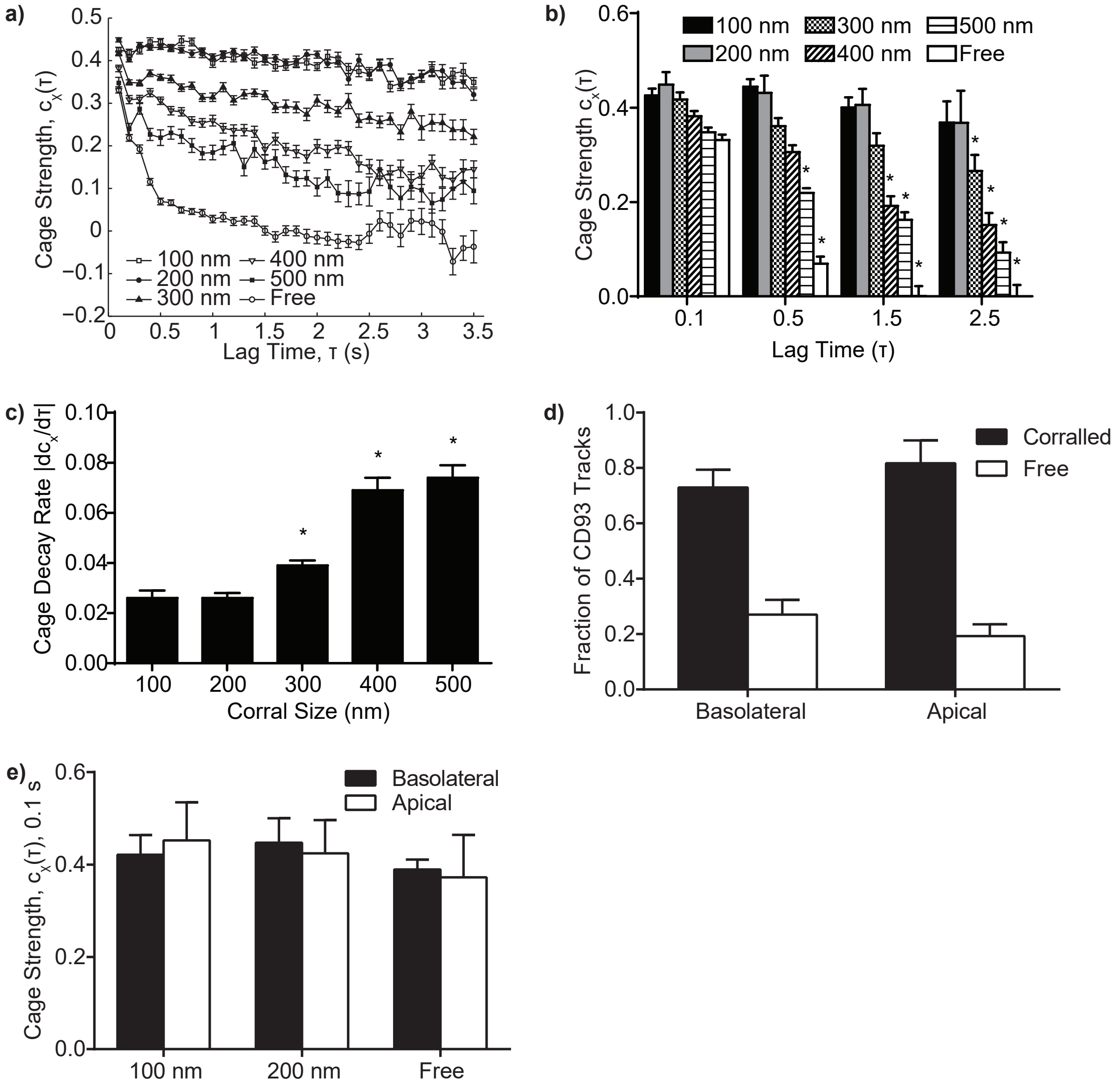

Supplemental Figure 2

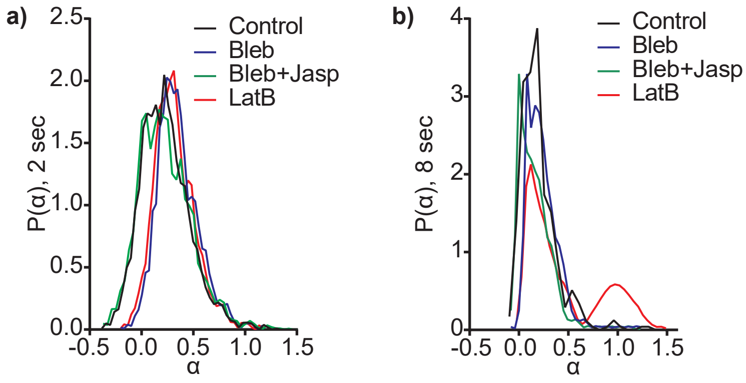

Supplemental Figure 3

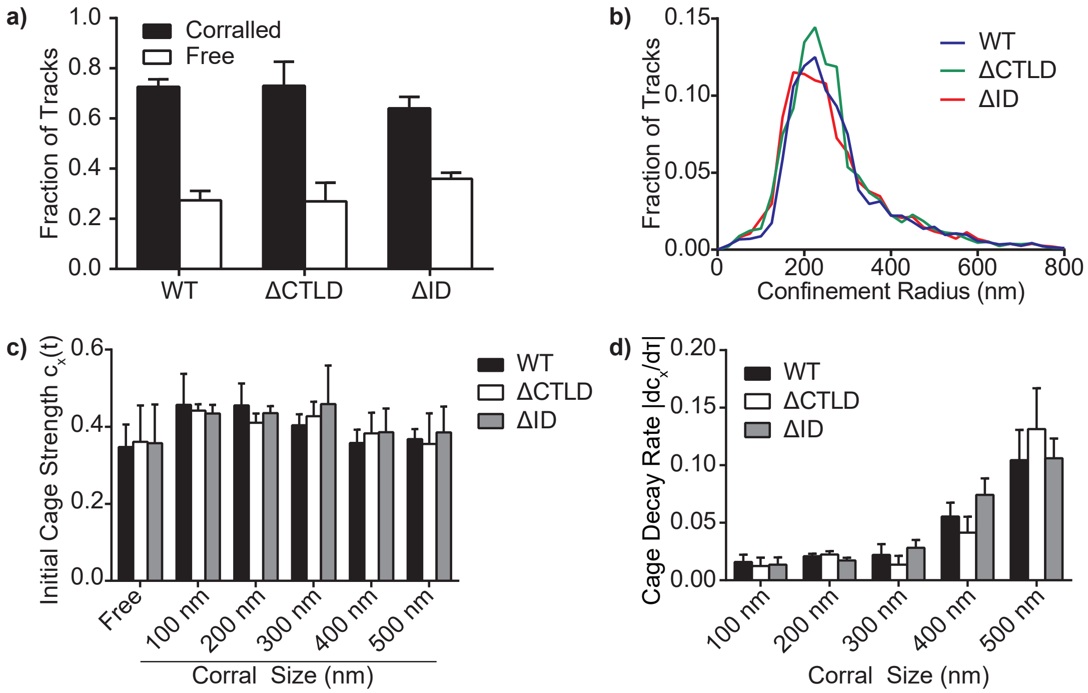

Supplemental Figure 4

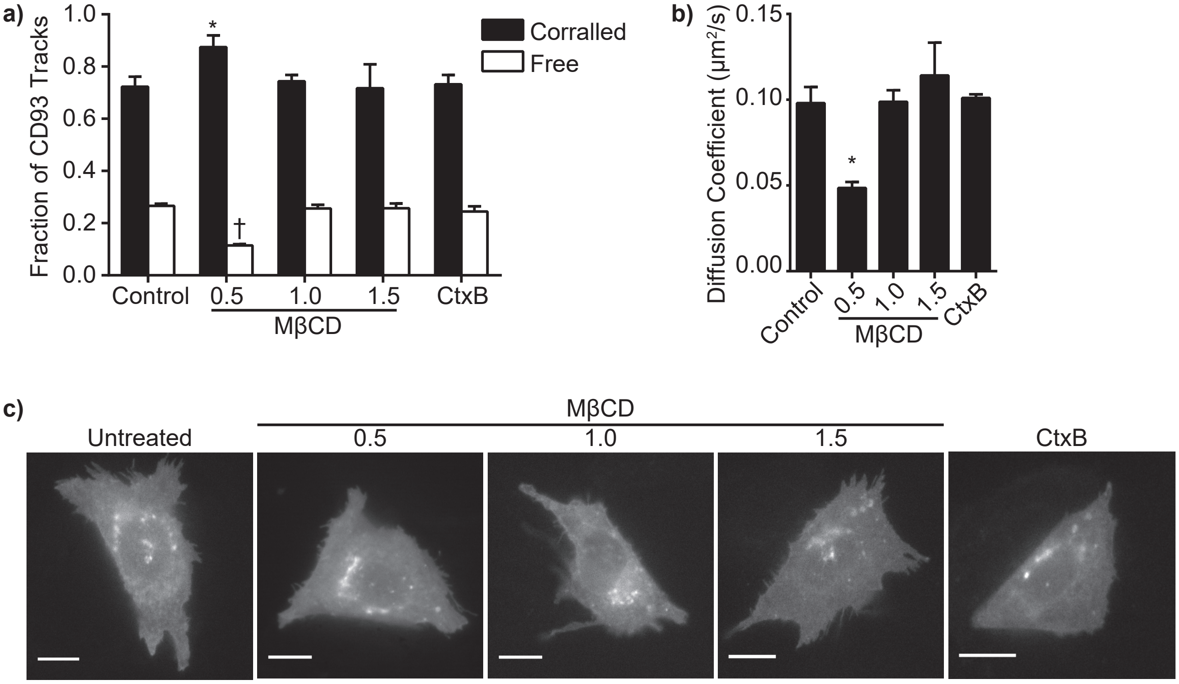

Supplemental Figure 5
